# Flow Cytometry: From Experimental Design to Its Application in the Diagnosis and Monitoring of Respiratory Diseases

**DOI:** 10.3390/ijms21228830

**Published:** 2020-11-22

**Authors:** Julio Flores-Gonzalez, Juan Carlos Cancino-Díaz, Leslie Chavez-Galan

**Affiliations:** 1 Laboratory of Integrative Immunology, Instituto Nacional de Enfermedades Respiratorias Ismael Cosio Villegas, 14080 Mexico City, Mexico; jfloresg1707@alumno.ipn.mx; 2 Laboratory of Immunomicrobiology, Microbiology Department of Escuela Nacional de Ciencias Biológicas, Instituto Politécnico Nacional, 11350 Mexico City, Mexico; jccancinodiaz@hotmail.com

**Keywords:** flow cytometry, experimental design, quality control, respiratory diseases, diagnostic, clinical follow-up

## Abstract

Recent advances in the field of flow cytometry (FCM) have highlighted the importance of incorporating it as a basic analysis tool in laboratories. FCM not only allows the identification of cell subpopulations by detecting the expression of molecules in the cell membrane or cytoplasm, but it can also quantify and identify soluble molecules. The proper functioning of the FCM requires six fundamental systems, from those related to the transport of events to the systems dedicated to the analysis of information. In this review, we have identified the main considerations that every FCM user must know for an optimal antibody panel design, the quality systems that must govern the FCM protocols to guarantee reproducible results in research or clinical laboratories. Finally, we have introduced the current evidence that highlights the relevance of FCM in the investigation and clinical diagnosis of respiratory diseases, establishing important advances in the basic and clinical study of diseases as old as Tuberculosis along with the recent proposals for the monitoring and classification of patients infected with the new SARS-CoV2 virus.

## 1. Introduction

The quantitative analysis of blood cells began in 1873 with the development of the hemocytometer, designed by Louis–Charles Malassez; subsequently, the cellular photoelectric counters made it possible to identify “individual events” smaller than 10 microns in single-cell suspension [1,2,3]. Subsequently, Crossland-Taylor developed the “hydrodynamic focusing” technology, which allowed automated systems for greater speed of analysis in cell counting; and finally, the detection system based on the electrical conductance of cells suspended in a conductive fluid, developed by Wallace H. Coulter, was integrated [4,5].

Flow cytometry (FCM) arises with the design of the cell spectrophotometer, which makes it possible to measure both the content of nucleic acids and the size of the analyzed cells [6]. For the development of FCM, the inkjet system of the printers was incorporated into the Coulter technology, allowing to generate a system of micro-drops in continuous fluid, which favoured the basic principle of cell separation known as Fluorescence-Activated Cell Sorting (FACS) [7,8].

The use of FCM in clinical practice required the development of (1) monoclonal antibodies (mAb), attributed to Cesar Milstein, Georges Köhler and Niels Jerne and, (2) molecules with intrinsic excitation and emission capacity at different wavelengths (fluorophores); since the former is required to be chemically coupled to fluorescent molecules to be detected by FCM [9,10,11].

**Figure 1:**
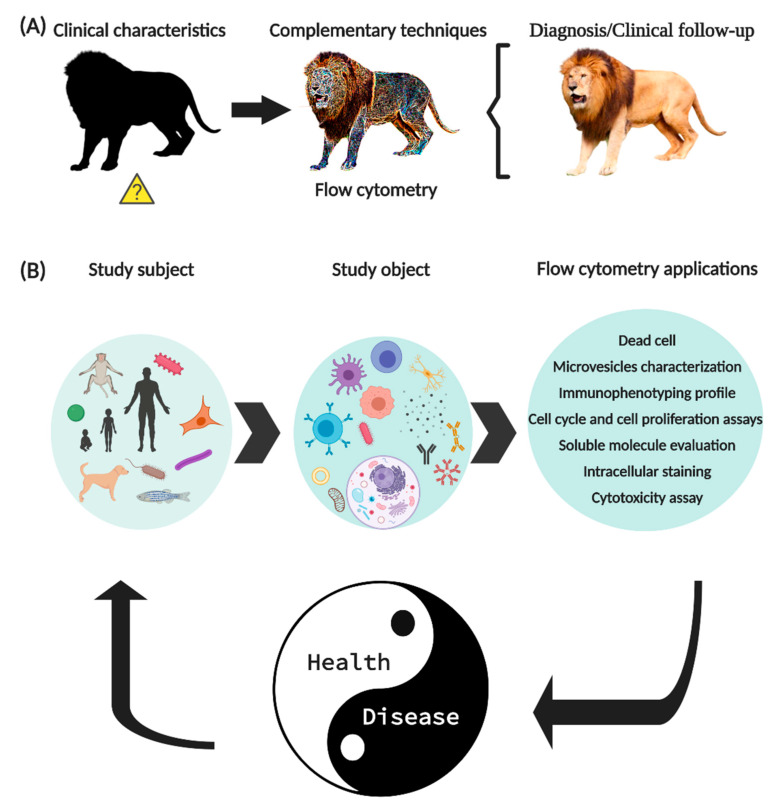
New generation flow cytometry applications. (**A**) FCM is a tool widely used in the clinic to support the diagnosis and clinical follow-up of patients. (**B**) The use of FCM is not limited to humans, its application allows to identify molecules expressed on the surface or intracellular of circulating cells, soluble molecules, signaling pathways, intracellular organelles, genetic material, and even microorganisms. In summary, FCM has allowed important improvements in basic research, as well as established parameters that help to diagnose and monitor various diseases.

Currently, the “International Society for Advancement of Cytometry (ISAC)” and the “International Clinical Cytometry Society (ICCS)”, are the institutions that dictate the correct standardization of techniques and equipment for FCM, to generate effective and reproducible results. FCM has established itself as one of the main tools to support the diagnosis and clinical monitoring of diseases since it allows the monitoring of the expression of specific cell markers (Figure 1A).

## 2. Basic Systems for the Operation of the Flow Cytometer

The expression “flow cytometry” is composed of the Greek ‘κντοζ (cell)’, ‘μετρον (measurement)’ and from the English word ‘flow’. FCM is a technique that allows evaluating events (commonly cells) in suspension, through the use of mAb coupled to fluorochromes (mAb-F). In FCM, the subject of the study is not limited to humans, since other animal species can also be studied, including bacteria. The use of FCM is diverse, it allows to identify cell’s subpopulation utilizing the expression of molecules in the cell membrane, cytoplasm or nucleus, besides soluble proteins (cytokines, chemokines, growth factors, etc.), extracellular vesicles, antibodies, etc. (Figure 1B).

The proper functioning of FCM is the conjunction of six fundamental systems, which involve different areas of science such as fluid physics, optics, electronics, and computing [12]; these systems are described below.

### 2.1. Fluid System

**Figure 2:**
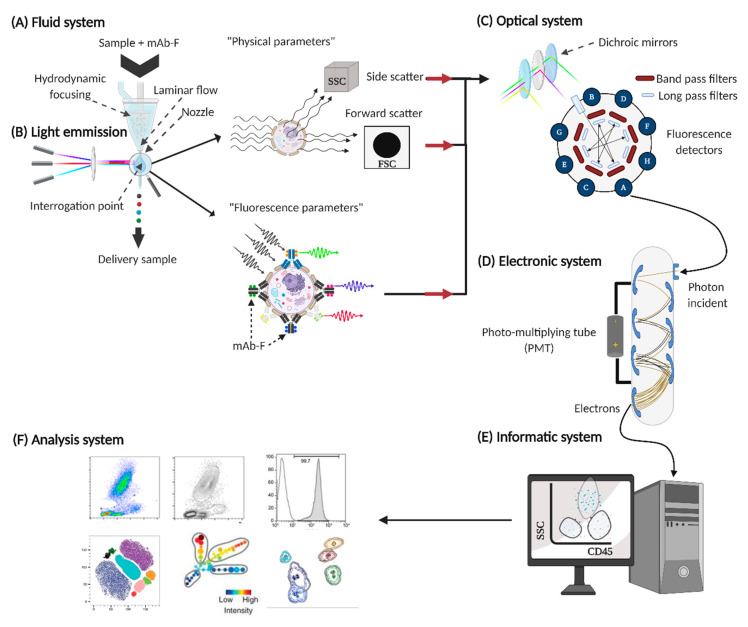
Fundamental systems in flow cytometers. For the proper functioning of the flow cytometer, six basic systems are required: (**A**) fluid system, (**B**) source of light emission, (**C**) optical system, (**D**) electronic system, (**E**) informatic system, and (**F**) analysis system.

The sample is prepared in suspension to favour the individual transport of the events to be analyzed (Figure 2A). At the sample interrogation point, the sheath fluid (saline) separates and aligns events (cells or molecules) individually for analysis [13]. In FCM, events can be identified from 0.5 µm (cell subpopulations) to less than 0.2 µm (extracellular vesicles, exosomes, microparticles) [14,15,16].

The nozzle controls the direction of the fluid and allows the alignment of events before moving to the interrogation point. The literature has suggested that the size of the nozzle to be used should be five times greater than the diameter of the event to be analyzed [17], however, it is important to standardize the use of the nozzle, prioritizing the needs of the analysis. Thus, the 70 μm nozzle and pressure of 70 pounds-force per square inches (psi) are suitable to analyze the blood circulation cells, bacteria, extracellular vesicles or quantification of molecules and soluble mediators [14,18]. Large cell analysis (cell lines, solid tissue cancer cells, plasma cells, dendritic cells) require low pressures with larger nozzle size, for example, a nozzle of 85, 100 and 120 µm with a system pressure of 45, 20 and 10 psi, respectively [18]. 

Once the appropriate nozzle is selected, drops are generated as a classification unit; the higher the number of drops, the faster the sorting speed and the shorter the time. For example, with the 70 μm nozzle, the frequency of classification units can generate up to 90,000 drops per second (90 kHz), which allows the analysis of approximately 30,000 particles/second (103–104 cells/second) [18,19].

### 2.2. Source of Light Emission 

Cytometers record light emission patterns within the visible electromagnetic spectrum, which ranges from ~350 to ~800 nm; Although the equipment can detect a greater range of the electromagnetic spectrum, the use of higher energy lengths (gamma rays, X-rays) causes the breakage of any type of covalent bond within the biological systems, and lower energy waves (microwaves, waves radio) do not create enough fluoresce [20,21].

**Figure 3:**
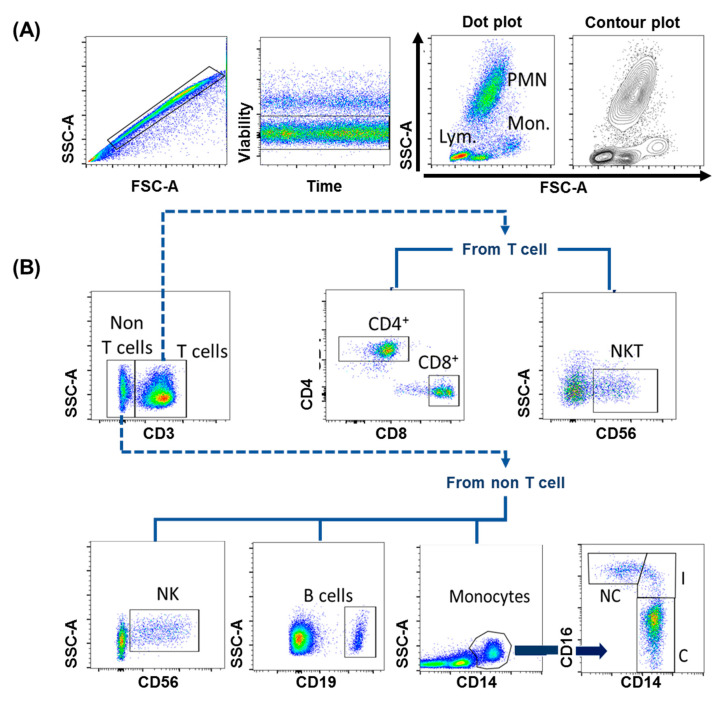
Mononuclear cells of blood circulation, evaluated by FCM. Peripheral blood mononuclear cells (PBMC) were labelled with mAb-F, targeting the CD56, CD3, CD4, CD8, CD19, CD14, and CD16 molecules, and a viability marker. Next, all events were acquired from FACSAria II cytometer. (**A**) The use of a viability marker allows to limit the analysis to living cells, simply, physical parameters are obtained, such as FSC and SSC, to identify, in general, the region of lymphocytes (Lym), monocytes (Mon) and polymorphonuclear cells (PMN). (**B**) The use of mAb, linked to fluorochromes, makes it possible to quantify “n” fluorescences that represent the evaluation of a molecule. In our example, the expression of CD3 events distinguishes two populations, T and non-T cells. The expression of CD56 in non-T events allows us to identify Natural Killer (NK) cells, and the expression of CD19 allows us to identify B lymphocytes. To identify monocytes, the expression of CD14 is evaluated, where the analysis CD14 vs CD16 allows us to identify the three best-characterized subpopulations of monocytes: classical (c), intermediate (I) and non-classical (NC). In T events (total lymphocytes), the expression of CD4 and CD8 allows the identification of CD4 (T helper) and CD8 (cytotoxic T) lymphocytes. Finally, the expression of CD56 in CD3+ cells makes it possible to identify Natural Killer T (NKT) cells.

At the interrogation point, a “laser” is impinged on the event to be analyzed, the electrons of the fluorochrome that is bound to the mAb are stimulated, and energy (light) is released at a different wavelength (Figure 2B) [22]. This phenomenon allows to evaluate the:

1. Physical parameters, such as size (FSC, forward scatter), this is associated with interference in the horizontal path of visible light, as well as cell complexity (SSC, side scatter), which is associated with the change in the refractive index in every interface of the analyzed event. With these basic parameters, the region of lymphocytes, monocytes and polymorphonuclear cells can be generally identified (Figure 3A).
2. Fluorescence parameters, quantify “n” fluorescences (depends on the capacity of each cytometer), allowing to obtain the percentage of positive events for a molecule (cell frequencies), and also to obtain the arbitrary unit called mean fluorescence intensity (MFI), which indirectly indicates the number of molecules expressed on the surface of an event [23].

### 2.3. Optic System

It allows to collect the light signals generated at the interrogation point, which are then directed to detectors using fibre optic cables and glass optical filters (Figure 2C):

1. Long pass (LP) transmits photons with a wavelength greater than that specified, for example, the LP 600 filter transmits light signals with a wavelength equal to or greater than 600 nm.
2. Short pass (SP) transmit photons with a wavelength shorter than specified, for example, SP 600 filter transmits signals with a wavelength shorter than 600 nm [24].
3. Band pass (BP) transmits light signals within the specified wavelength range, for example, the BP 525/50 filter, which allows light signals to pass between 500–550 nm.
4. Dichroic mirrors (LP or SP), placed at a 45° angle to the incident light, reflecting non-transmitted light at a 90° angle to improve the transmission of wavelengths to specific detectors [25].

### 2.4. Electronic System

Once all the signals of specific wavelength have been captured, they are transformed into an electrical signal, converting the photons into electrons through photodetectors called channels (Figure 2D); subsequently, the signal is amplified by photomultiplier tubes (PMTs) [26]. In the end, the generated information acquires digital values, which are arranged in files called flow cytometry standard (FCS) to be analyzed in computer systems.

The way to express the analyzed properties at the interrogation point is through analogue-digital conversion (ADC), generating a binary language or bits. For example, a FACScalibur cytometer has a 10-bit ADC, which means that 1024 values can be assigned to the generated electronic pulse ($2^{10})$. While a FACsAria II cytometer has an 18-bit ADC and generates up to 262144 values ($2^{18}$), that is, the higher the number of bits corresponds to a higher resolution of the information [27].

### 2.5. Informatic System

The function of the informatic system is to facilitate the reading and graphic interpretation of the FCS files generated from the analyzed events (Figure 2E) [28].

### 2.6. Analysis System

A variety of software for the analysis of FCM data are existing, which allows establishing different analysis strategies through graphic representations of our data set (Figure 2F) [29,30]. 

The most used graph is the dot plot; this represents the total distribution of the captured events, considering that each point of the graph represents an evaluated event. There are two forms of representation: (1) axes with logarithmic scales, to present fluorescence values that differ between 1000 to 100,000 times the increase. The null expression is below logarithm 101 and they are used when the expression of the analyzed parameter is homogeneous and strong in intensity; by its nature, the logarithmic scale hides values with low fluorescence expression. (2) Axes with a bi-exponential scale, which present logarithmic values between 0 and 100 on the same axis, allow to visualize the complete distribution of the analyzed events [31].

The contour plot shows the relative intensity distribution by density in circumferences; events with little variation in the evaluated parameter are placed in the centre and events with greater variation are placed outside [32].

Histograms allow observing the uni-parametric variations on the X-axis and the number of events on the Y-axis [33]. 

FCM evaluations commonly encompass the study from eight parameters and combinations of 28 dot plots up to 50 parameters simultaneously in the same sample. There is also the possibility of generating analysis algorithms of 12–30 simultaneous parameters or grouped in different tubes, where common mAb-F are used to identify general populations, and additionally mAb-F to characterize subpopulations of interest. These latter forms of analysis can generate a minimum of 66 graphical representations (for example, dot plot), so mono or bi-parametric plots are not very effective. To solve this problem, the new analysis programs have integrated multivariate analysis, which groups events with similar characteristics to show graphs that represent homogeneous groups in distribution and expression of parameters [34].

## 3. Basic Considerations to Perform Flow Cytometry 

To date, more than 400 mAbs have been standardized and approved for use in research and clinical diagnosis in human leukocytes [35]. When considering FCM as a work tool, we must start from basic questions to establish an adequate experimental design that suits our interests, for example:

1. Related to the object of study: Will cells, soluble components, functional processes (in vitro activation) be evaluated?
2. Related to the staining protocol: What type of sample is it? Do I need to identify surface marks, intracellular, phosphoproteins, etc? Will the acquisition of the samples be immediate or do I need to preserve them?
3. Equipment related: What equipment is available? How many types of lasers do you have attached? What detectors does it have?
4. Related to fluorescence: How much do the emission signals of the selected fluorochromes overlap?
5. Related to the analysis system: What analysis program is available?

It is recommended that for the selection of the fluorochrome that will be coupled to the mAb, the intensity of expression of the molecule to be evaluated is considered; thus, with regard to molecules with abundant expression, it is preferable to use low-bright fluorochromes such as those of the blue laser (488 nm) and red (633 nm), while for molecules with minimal or unknown expression, it is preferable to use bright fluorochromes such as those excited by the violet laser (405 nm) [36]. However, each commercial house establishes its own rules for the use of its mAbs and fluorochromes, so it is essential to read their recommendations.

Another consideration for choosing fluorochrome is to avoid selecting those that fluoresce at similar wavelengths, for example, the emission spectrum of fluorochrome called phycoerythrin cyanine 5 spans ~637–705 with a maximum emission at 667 nm; because the fluorochrome called allophycocyanin (APC) emits 660 nm wavelength, the detector for APC can recognize the signal and quantify false positives. To avoid these errors, it is essential to visualize the excitation and emission range of the fluorochromes to be used. Currently, almost all commercial houses provide a tool called spectra viewer, and it is very useful to avoid using mAb-F whose fluorochromes fluoresce at a similar wavelength [23].

## 4. Quality Management Systems in Flow Cytometry Assays

The applications of FCM are aimed at evaluating the expression pattern of molecules in health and disease, based on the biological principle that there are both phenotypic and functional cellular alterations in the immunopathogenesis of disease [37].

Currently, FCM serves as a tool for the diagnosis and clinical monitoring of pathologies of various origins; including oncological, immunodeficiencies or infections, it is essential not to compromise the obtained results for the proper processing of the samples, for example, it is recommended that peripheral blood samples from patients with sepsis be subjected to bi-hemolysis processes, to avoid problems of specificity of mAb-F [38,39].

The use of FCM requires applying a comprehensive quality management system throughout the process, which is explained in the subsequent sections:

### 4.1. Pre-Analytical Phase 

This phase establishes the criteria for taking and processing the samples to be analyzed, considering which are the cell populations or molecules of interest [40]. In this phase, characteristics such as the origin of the sample (peripheral blood, bone marrow, cell cultures, etc.) and amount of sample to be used (microliters, millilitres) must be established; for instance, when blood is taked, the user should determinate if they need only the cell fraction, serum or plasma; because also should be to determinate a type of anticoagulant that does not interfere with the analysis of the sample (EDTA, heparin, etc.). Additionally, the appropriate time between obtaining the sample and its analysis must be identified, as well as the mechanism for transporting and preserving the specimen; finally, in the case of cryopreservation, the optimal conditions must also be established.

### 4.2. Analytical Phase 

In this phase is establishes the characteristics to optimize and reproduce the assay through experimental controls that guarantee the reproducibility of the results [41]. It includes:

1. Internal quality control: useful for monitoring the variations of the variables over time using Leving–Jennings graphs; this establishes the standard deviation of the signal from each fluorescence detector.
2. Autofluorescence control: allows to know the natural fluorescence pattern of the analyzed events; the sample is subjected to all the processes, except the staining protocol. The result will be the inherent characteristic of the excitation and emission of the molecules that make up the analyzed event (background fluorescence).
3. Fluorescence minus one (FMO): These controls contain all the mAb-F used in the panel, except for one, which is relevant to the molecular markers to be studied. It helps to explain propagation error in the empty detector by other interfering fluorescence signals (compensation problems) [42].
4. Isotype control: This control allows to differentiate the non-specific binding of the fragment crystallizable region of the antibodies and the aggregation of fluorescent molecules by excess. Due to the variability of the manufacturing processes, it is impossible to have the ideal isotype control [42]; currently, its use is no longer widely recommended.
5. Viability staining: It allows limiting the analysis of the molecules of interest in viable cells, distinguishable by evaluating cell permeability through dyes that enter the cell and indirectly indicate cell death (Figure 3A) [43].
6. Staining control: refers to the titration of the mAb-F to know the optimal concentration of mAb-F that saturates the available sites of the antigen to be detected, thereby avoiding the presence of false positives due to excess mAb-F [42].
7. Background noise: phenomenon attributed to photons with random emission from cell fragments or inadequate compensation. The value of the signal that is considered a “normal” event (threshold) is limited, based on the size and complexity of the cellular event of interest.

### 4.3. Post-Analytical Phase 

In this phase is established the appropriate representation of the results, for example, to identify populations of cells in the periphery (Figure 3B), which allows the preparation of reports with a simple and practical interpretation for health professionals and ensures that the information is comparable with the results obtained in various flow cytometry equipment [44].

## 5. Use of Flow Cytometry in the Diagnosis of Respiratory Diseases

The respiratory diseases are the most common causes of death worldwide [45]. The Forum of International Respiratory Societies (FIRS) defines respiratory disease as those that “affect the airway and lungs, impacting lung capacity or gas distribution in our body”, and the five main ones are chronic obstructive pulmonary disease (COPD), asthma, acute lower respiratory tract infections (AILRT), tuberculosis (TB), and lung cancer [46].

Techniques such as single-cell RNA sequencing, mass cytometry and FCM have been essential to describe pathophysiological mechanisms and to search for diagnostic markers in respiratory diseases [47]. In the next sections, we have demonstrated the experimental evidence that exists on the importance of using FCM as a tool for monitoring and diagnosing respiratory diseases.

### 5.1. COPD

It is the third leading cause of death worldwide; poor air quality and smoking are risk factors highly associated with this disease. As part of the pathophysiology, the circulating release of inflammatory mediators promotes the activation and recruitment of circulating cells to lung tissue [48].

**Figure 4:**
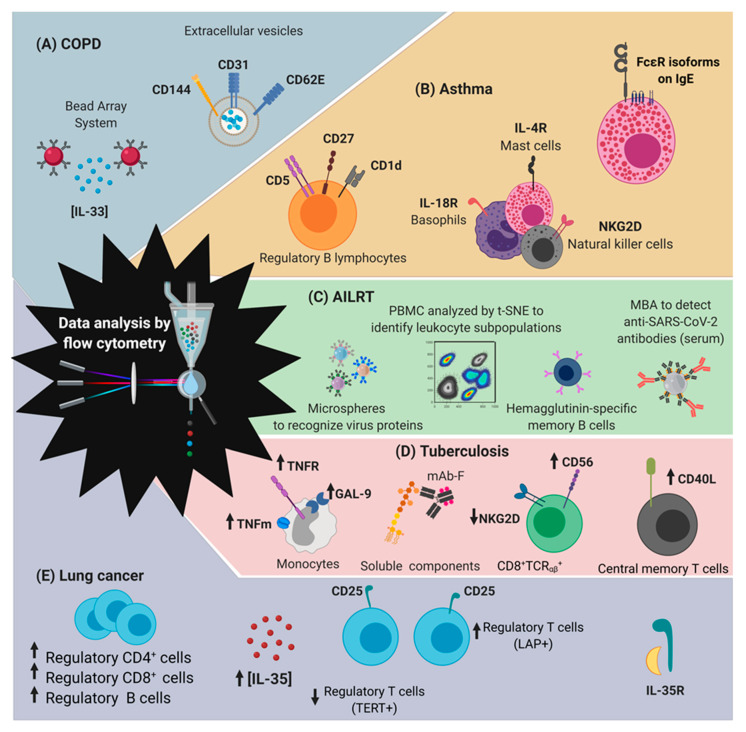
Developments in the diagnosis of respiratory diseases obtained by flow cytometry (FCM). FCM has made it possible to establish parameters for the diagnosis and study of the five main respiratory diseases: (**A**) chronic obstructive pulmonary disease (COPD), (**B**) asthma, (**C**) acute lower respiratory tract infections (AILRT), (**D**) tuberculosis and (**E**) lung cancer. Abbreviations: t-SNE, high-dimensional unbiased stoichiometric analysis; PBMC, peripheral mononuclear cells; MBA, microsphere-based antibody assay; mAB-F, Monoclonal antibodies coupled to fluorochromes; GAL-9, Galectine 9; LAP, Latency Associated Peptide; TERT, telomerase reactivity.

Using FCM as a tool, it has been described that COPD patients have high concentrations of IL-33 (alarmin) in the bloodstream, favouring the generation of autoantibodies directed against type II alveolar epithelial cells, and consequently there is damage to lung tissue [49]. Furthermore, FCM has also made it possible to identify extracellular vesicles (EVs) identified by the molecules CD144^+^ (VE-cadherin), CD31^+^ (PECAM-1) and CD62E^+^ (E-selectin), and collectively, from the pulmonary capillaries of COPD patients; high levels of EVs can occur in plasma, even before clinical symptoms [50]. The finding of these vesicles would represent an advance in the search for diagnostic and monitoring markers for specific COPD for the follow-up of patients with COPD (Figure 4A).

### 5.2. Asthma

It is a chronic inflammatory disease of the airways, in its pathophysiology, type I hypersensitivity processes are involved, and in industrialized countries, high concentrations of particulate matter that is suspended in the air, acts as an adjuvant in the mechanisms and triggers asthma [51].

Basophils, monocytes and plasmacytoid dendritic cells from asthmatic children living in industrialized regions have recently been described as having an increase in the expression of the specific receptor IgE –FcεRIα [52]. As part of the clinical follow-up, Wiest et al. [53] described that patients with asthma have decreased lymphocyte B CD5^+^ and CD1d^+^CD5^+^ frequency, but an increase in cells B CD27^+^, suggesting that monitoring the expression of CD5, CD1d and CD27 in B cell subpopulations is useful in the clinical follow-up of people with asthma. Together, these results have made it possible to establish clinical correlations that identify people with greater susceptibility to the development of asthmatic allergy and open up new options in the search for therapeutic targets.

The Global Initiative for Asthma (GINA) has accepted as a prognostic factor in the clinical follow-up in children with therapy-resistant asthma (STRA), the identification by FCM of invariant (V_α24_J_α18_) NKT cells, where the percentage increase of said subpopulation correlates with the clinical complications of asthma [54]. By FCM and with the use of mAbs that recognize specific polymorphic variations, alterations have been identified in the receptors: lectin type C (for example, NKG2D); vitamin D (VDR); IL-4α; and IL- 18 on cytotoxic cells, mast cells and basophils. These alterations correlate with the predisposition to the development of bronchiolitis in animal models [55,56,57,58]. Thus, polymorphic variations in cells of the immune response and characterization of cell subpopulations have been established by FCM for the study of asthma (Figure 4B).

### 5.3. Acute Infections of the Lower Respiratory Tract

The morbidity and mortality rates associated with respiratory infections by viruses such as H1N1, Parainfluenza, rhinovirus, influenza B, as well as the new SARS-CoV-2 virus, affect a large percentage of the world population from infants to the elderly [59,60,61].

Swieboda et al. [62] have proposed an array for FCM, including 16 antibodies and 14 fluorochromes, to identify the three subpopulations of innate lymphoid cells, NK cells, monocytes, NKT cells, and granulocytes (neutrophils and eosinophils) by high-dimensional unbiased stoichiometric analysis (t-SNE), in addition to generating cell maps to identify variations related to a particular antiviral immune response during acute respiratory tract infections.

Under influenza context, it has been suggested that the use of microsphere-based flow cytometry immunoassay allows the functional characterization of influenza viruses, although mAb recognizes virus proteins and distinguishes between the inactivated virus and influenza virions; moreover, using this tool also allows the functional assessment of relative receptor affinity [63,64]. Recently, through FCM, hemagglutinin-specific memory B cells that express IgG1 were identified in previously vaccinated healthy adults; this result provides alternatives to evaluate the immune response as a response to booster vaccination or as a complementary test to diagnosis [65].

The analysis by t-SNE also is proposed to be use used for the study of leukocytes from patients infected by the SARS-CoV-2 virus, since it is suggested that patients infected with SARS-CoV-2 present higher percentages of naive T cells and lower percentages of memory T cells [66]. Altogether, clinical variables and alterations in the cells of the immune system, evaluated by t-SNE, have established three profiles of patients infected with SARS-CoV-2: (1) They present robust activation and proliferation of CD4^+^ T lymphocytes together with CD8^+^ T lymphocytes highly activated and exhausted; (2) discrete response of CD4+ T lymphocytes and CD8^+^ T lymphocytes with high expression of t-bet and memory B cells; and 3) lymphocytes deficient for the activation process and therefore inefficient to face infection [67]. This classification of patients infected with SARS-CoV-2 has an important impact on the implementation of appropriate therapies based on the immunological profile of each patient.

In another way, in order to improve the sensitivity and specificity of the currently available COVID-19 antibody tests, Fong et al. [68] proposed the use of the methodology called microsphere-based antibody assay (MBA); authors suggest that MBA is a suitable and straightforward test for clinical microbiology laboratory; it is useful to determinate anti-SARS-CoV-2 antibodies. MBA, compared to the classic ELISA assay, showed a specificity of 100% and seropositive rate for convalescent COVID-19 patients of 89.8%. Thus, it is proposed as a new diagnosis test of SARS-CoV-2 infection, even in patients with low viral load (Figure 4C).

### 5.4. Tuberculosis

Tuberculosis (TB) is an infectious disease caused by the bacillus *Mycobacterium tuberculosis* (Mtb). In 2018, the World Health Organization (WHO) reported nearly 10 million TB patients, 89% of the cases occurred in men and women over 15 years of age and 11% in infants [69].

Mtb has virulence factors of both protein and lipid origin. Through FCM, it was shown that monocytes exposed to the glycolipid lipoarabinomannan (LAM), coming from the Mtb cell wall, affect their macrophage maturation process; phenotypically they have decreased expression of the molecules Galectin 9, TLR (Toll-like receptors) 2 and 4, CD68, CD33, and CD86, suggesting that macrophages generated under these conditions are poorly functional and efficient to control the growth of Mtb [70,71].

It was recently shown that TB patients have a higher frequency of cells CD8^+^TCR_αβ_^+^NKG2D^dim^CD56^high^, and these cells have increased cytotoxic function [72]. The use of FCM has also allowed the identification of non-classical cell populations such as monocytes and macrophages that express the CD3/TCR_αβ_ complex in response to mycobacterial infections. This cell population increases both at the systemic level and at the site of infection; apparently its presence is regulated by a TNF-dependent pathway, carrying out phagocytosis processes, and they are an important source of pro-inflammatory cytokines [73,74,75]. The increase in this cell population suggests that its frequency can be monitored in TB patients as a biomarker, showing the relevance of the use of FCM in the search for new markers of cell function in the context of TB.

Recently, Esteves et al. [76] identified new potential markers to establish an analysis algorithm that allows discriminating between patients with active TB and latent TB by analyzing the expression of CD154 (CD40L) in central memory CD4 T lymphocytes (TCM) as the best candidate, since it has been observed that this population expands after activation with Mtb, contributing to CD27 and CD45RO expression in TCM. In the same sense, the expression of CD161 in T cells has been observed as a potential marker to distinguish the latent infection state, whose sensitivity of 74% and specificity of 86% could be sufficient to integrate it into the diagnosis together with other classic tuberculosis tests [77].

FCM has allowed the identification of cellular phenotypes that modulate their activation against bacterial infections. Evaluating the cellular heterogeneity present during Mtb infection will help to establish biomarkers that allow better follow-ups in TB patients (Figure 4D).

### 5.5. Lung Cancer

Only 16% of patients with lung cancer are diagnosed in the early stages of the disease, 85% of the cases are of the non-small cell type (NSCLC), which promote the evasion strategy of the immune system to generate immunosuppressive environments to systemic level [78]. The expression of programmed cell death protein 1 (PD-1), by FCM, has been essential to establishing the initiation of therapy with blocking mAbs of PD-1 or PD-L1, and thus, improve the response of the immune system against tumour cells and improve the quality of life of patients [79,80].

In recent years, the description of cell populations with an anti-inflammatory function such as TCD4^+^_reg_, CD8^+^_reg_ or B_reg_ and their correlation to circulatory concentrations of IL-35 have been associated with the evolution of treatments in lung cancer [81].

Kotsakis et al. [82] have observed differences in the frequencies of naive, effector and terminal subpopulations in cells T_reg_CD4^+^CD25^high^ circulating in patients with NSCLC, raising the possibility of generating prognostic and predictive value, as well as therapeutic targets by proving the ability to suppress the activation of CD4^+^ lymphocytes producing IFN-γ. In addition, it has been observed that clone-specific Treg cells can potentially contribute to the reduction of CD4^+^ cells with telomerase reactivity (TERT) and the monitoring in Latency Associated Peptide (LAP)-TGF-β specific Treg cells that participate in the decrease of cellular activity against aberrant cells, which together could complement the analysis strategy and increase the sensitivity to the early diagnosis of lung cancer [83,84]. In general, these results generate new alternatives to the clinical follow-up of patients and whose non-invasive methodology entails rapid sample processing.

In the same context, Liu et al. [85] have observed the importance of the loss of expression of CD28 in CD8^+^ lymphocytes with the progress to advanced stages of lung cancer. In summary, the study of Interleukin (IL)-35 signalling mechanisms, as well as the expression of the IL-12p35 and IL-27 receptors involved in the acquisition of circulating cells towards tolerant phenotypes could represent a window to identify cell populations that have been correlated with early and follow-up diagnoses in the evolution of lung cancer [81]. Figure 4E summarizes the main findings, identified by FCM, for better diagnosis and follow-up of patients with lung cancer.

## 6. Advantages and Limitations of Conventional Methods, Compared to FCM, in the Diagnosis of Respiratory Diseases

Currently, specialized agencies in public health such as the WHO and the centers for disease control and prevention, establish and approve guidelines for the diagnostic and follow-up of different pathologies, including those of respiratory origin. Table 1 shows a summary of the conventional diagnostic method to respiratory diseases, and it is contrasted with the alternatives that could be evaluated by FCM.

As we discussed above, FCM is a technique that has been shown to be useful to improve the diagnosis and monitoring of respiratory diseases, majorly in those pathologies where the conventional technique for diagnostics (provided in the guidelines) has inconveniences like low sensitivity and specificity, highly invasive technique to obtain the tissue sample, expensive, and long time to deliver results, among others. The measuring of molecules by FCM usually is implemented in peripheral mononuclear cells or serum; this means, a blood sample is sufficient to develop the quantification of molecules by FCM. Moreover, although this technique requests sophisticated equipment and special capacitation to use it, FCM results are delivered in a short time, and they are relatively easy to interpret. Finally, flow cytometer is a stable machine that has the potential to implement a point-of-care test.

Guidelines provided by European Respiratory Society (ERS), American Thoracic Society (ATS) and Global Initiative for Chronic Obstructive Lung Disease (GOLD) recommend the use of lung function tests and imaging tests in the diagnosis of respiratory diseases [86,87]. For instance, spirometry test is relevant for COPD diagnosis, and though it is an excellent tool to support a clinical decision, to have quality spirometric measurements, a consensus among manufacturers, clinicians, operators, and researchers is requested in order to improve the accuracy and precision of the test [88,89,90]. 

Methods used for TB diagnosis also show limitations, for instance, culture-based methods need a long time to deliver the result, and this delays the start of the treatment and another test, as the tuberculin skin test has low specificity [91]. Currently, serological and nucleic acid amplification tests are useful to identify influenza or SARS-CoV-2 infection. However, RT-PCR test is expensive, and although it is highly specific and sensitive, there are reports of false-negatives and -positives that are majorly associated with the high homology between strains or because the test is detecting non-viable virus or cell debris; whereas, some serological test requests improve its sensibility and specificity [92]. 

Finally, biopsy is a method used for lung cancer diagnosis but is a highly invasive method to get the tissue sample. Moreover, it should be done by competent personal that request training for a long time.

## 7. Conclusions

Diseases that affect the respiratory system influence humans towards high mortality and morbidity, regardless of age, gender or social status. These conditions are subject to our lifestyle, geographic environment, and climate change.

In the history of humanity, diseases that arise with clinical characteristics of their pathophysiology have been described. Therefore, tools are needed to address the following criteria:

- Quick and specific diagnosis.
- Identification of clinical phases.
- Follow-up biomarkers to evaluate adequate response to treatments.
- Cost–benefit ratio.

Within the current clinical evaluation tools, special attention has been paid to the specificity and sensitivity for the diagnosis and follow-up of diseases offered by FCM. Since its inception, it has emerged as a tool for rapid and individualized interrogation of suspended events. Through continuous improvement in the basic principles of fluorochrome excitation and emission, monoclonal antibody production, and data analysis systems, today’s cytometrists have been equipped with powerful tools to extend our knowledge of biological systems.

**Figure 5:**
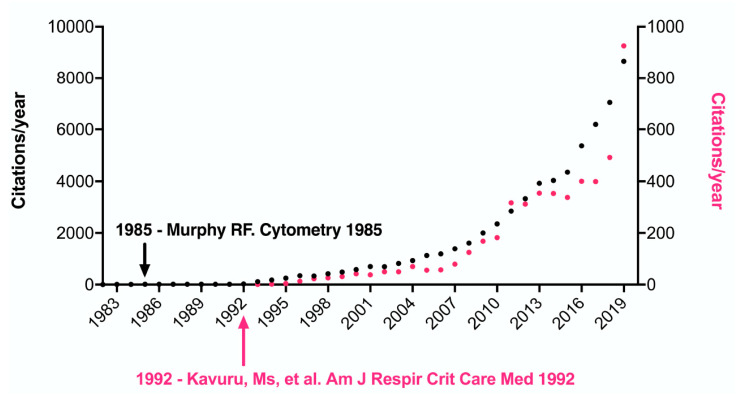
Citations on “Flow cytometry and respiratory diseases”. The annual number of citations in articles for the keyword ‘flow cytometry and respiratory diseases’ is represented by black dotted circles (Y-axis on the left side), and the number of citations per year for the keyword ‘flow cytometry and clinical diagnosis’ is presented by pink dotted circles (Y-axis on the right side), which have entered cytometry for diagnosis and follow-up since 1993. The data is generated from the Web of Science.

From 1985, when the first analysis algorithm was proposed with the evaluation of more than three molecules, the interpretation and analysis of the results remained quite challenging. Since 1993, the boom in FCM began in the diagnosis of respiratory diseases, as can be seen in Figure 5; the black dotted line shows the annual increase in the number of citations of articles when searching for the phrase “flow cytometry and respiratory disease” (Y-axis, left), showing (with the black arrow) the year of the first reported circulating cell analysis algorithm. While the dotted pink line indicates the citations of articles for the phrase “flow cytometry and clinical diagnosis” (Y-axis, right), thus showing that the use of FCM for diagnosis and monitoring of diseases worldwide has expanded.

In this sense, cytometry studies must undergo rigorous standardization processes, as well as further development of multicenter studies, which are the fundamental characteristics that a method must meet to establish quality clinical studies. FCM users must prioritize the development of adequate standardization protocols and normalize the study population to have a greater impact on the identification of cell populations.

The proper management of FCM should favour the creation of reliable databases that, when compared between study groups, lead to the best diagnosis and follow-up of patients under various treatment schemes (immunological, pharmacological, etc.).

## Figures and Tables

**Table 1:** Flow cytometry as an alternative in the diagnosis and monitoring of respiratory diseases.

Respiratory Disease		^[A]^Conventional Diagnostic and Monitoring Methods	Alternative to Evaluate by Flow Cytometry to Support the Diagnostics and Monitoring	References
**^[B]^** **COPD**		Spirometry test	IL-33 (alarmin)	[49]
			Extracellular vesicles (CD144^+^CD31^+^CD62E^+^)	[50]
**Asthma**		Skin test ^[C]^FeNO test Family history	IgE –FcεRIα expression on basophils, monocytes and plasmacytoid dendritic cells	[52]
			CD5, CD1d and CD27 expression on B cell	[53]
			NKT invariant cells (V_α24_J_α18_)	[54]
			NKG2D expression on NK cells	[55]
			CD8^+^IL-13^+^ T cell	[56]
			IL-4R expression on conversion from T_ireg_ to Th_17_	[57]
			IL- 18R expression on basophils and mast cells	[58]
**^[D]^** **AILRT**	**Influenza**	^[E]^RT-PCR Lung exploration	Cell maps to identify variations of PBMC based on ^[F]^t-SNE analysis	[62,66]
			Microsphere-based antibody assay	[63,64]
			Hemagglutinin-specific memory B cells	[65]
	**SARS-CoV-2**	^[G]^NAAT ^[E]^RT-PCR Serological testing	Cell maps to identify variations of T cells based on ^[F]^t-SNE analysis	[67]
			Microsphere-based antibody assay	[68]
**TB**		Clinical data ^[H]^IGRA ^[I]^TST ^[JI]^AFB Smear Mycobacterial Cultures ^[F]^NAAT	Galectin 9, TLR-2 and 4, CD68, CD33, and CD86 expression on macrophages and monocytes	[70,71]
			CD8+TCRαβ+NKG2DdimCD56high	[72]
			CD3/TCRαβ monocytes	[73,74,75]
			CD40L expression on T cells (central memory)	[76]
			CD161 expression on T cells	[77]
**Lung cancer**		Imaging tests Biopsy Lung function tests	PD-1 expression	[79,80]
			IL-35 on NSCLC	[81]
			T_reg_CD4^+^CD25^high^	[82]
			CD4^+^TERT^+^ / CD4^+^LAP-TGF-β^+^	[83,84]
			CD8^+^CD28^+^	[85]

Abbreviations: ^[A]^Conventional is defined as those established by WHO and CDC in their guidelines. ^[B]^Chronic obstructive pulmonary disease. ^[C]^Nitric Oxide test. ^[D]^Acute infections of the lower respiratory tract. ^[E]^Reverse transcription polymerase chain reaction. ^[F]^High-dimensional unbiased stoichiometric analysis. ^[G]^Nucleic Acid amplification test. ^[H]^The interferon gamma release assay test. ^[I]^Tuberculin skin tests. ^[J]^Acid-fast bacillus test.

## Abbreviations

AILRT	Acute lower respiratory tract infections
ATS	American Thoracic Society
COPD	Chronic obstructive pulmonary disease
ERS	European Respiratory Society
EVs	Extracellular vesicles
FCM	Flow Cytometry
FCS	Flow cytometry standard
FIRS	Forum of International Respiratory Societies
FMO	Fluorescence minus one
FSC	Forward scatter
GINA	Global Initiative for Asthma
GOLD	Chronic Obstructive Lung Disease
ICCS	International Clinical Cytometry Society
IL	Interleukin
ISAC	International Society for Advancement of Cytometry
LAM	Lipoarabinomannan
mAB	Monoclonal antibodies
mAB-F	Monoclonal antibodies coupled to fluorochromes
MFI	Mean fluorescence intensity
Mtb	*Mycobacterium tuberculosis*
NKG2D	Natural killer group 2 member D
NSCLC	Non-small cell type
PBMC	Peripheral blood mononuclear cells
SSC	Side scatter
STRA	Therapy-resistant asthma
TB	Tuberculosis
WHO	World Health Organization
